# Increased risk of chronic kidney disease in patients with rosacea: A nationwide population-based matched cohort study

**DOI:** 10.1371/journal.pone.0180446

**Published:** 2017-10-02

**Authors:** Hsien-Yi Chiu, Wen-Yen Huang, Chung-Han Ho, Jhi-Joung Wang, Sung-Jan Lin, Ya-Wen Hsu, Ping-Jen Chen

**Affiliations:** 1 Institute of Biomedical Engineering, College of Medicine and College of Engineering, National Taiwan University, Taipei, Taiwan; 2 Department of Dermatology, National Taiwan University Hospital Hsin-Chu Branch, Hsinchu, Taiwan; 3 Department of Dermatology, National Taiwan University Hospital and National Taiwan University College of Medicine, Taipei, Taiwan; 4 Department of Medical Research, Chi Mei Medical Center, Tainan, Taiwan; 5 Department of Hospital and Health Care Administration, Chia Nan University of Pharmacy and Science, Tainan, Taiwan; 6 Department of Pharmacy, Chia Nan University of Pharmacy and Science, Tainan, Taiwan; 7 Research Center for Developmental Biology and Regenerative Medicine, National Taiwan University, Taipei, Taiwan; 8 Department of Geriatrics and Gerontology, Chi-Mei Medical Center, Tainan, Taiwan; 9 Department of Family Medicine, Kaohsiung Medical University Hospital, Kaohsiung Medical University, Kaohsiung, Taiwan; University of Colorado Denver School of Medicine, UNITED STATES

## Abstract

**Background:**

Rosacea is a chronic inflammatory skin disorder. Inflammation and oxidative stress are involved in the etiopathogenesis of rosacea and chronic kidney disease (CKD). This study aimed to investigate the association between rosacea and CKD.

**Methods:**

This population-based cohort study identified 277 patients with rosacea in the Taiwan National Health Insurance Research Database during 2001–2005. These patients were matched for age, sex, and comorbidities with 2216 patients without rosacea. All subjects were individually followed-up for 8–12 years to identify those who subsequently developed CKD

**Results:**

The incidence rates of CKD per 1000 person-years were 16.02 in patients with rosacea and 10.63 in the non-rosacea reference population. After adjusting for other covariates and considering the competing risk of mortality, patients with rosacea remained at increased risk of CKD (adjusted sub-distribution hazard ratio (aSD-HR) 2.00; 95% confidence interval (CI) 1.05–3.82). The aSD-HRs (95% CI) for CKD were 1.82 (0.83–4.00) and 2.53 (1.11–5.75) for patients with mild and moderate-to-severe rosacea, respectively.

**Conclusions:**

Rosacea is an independent risk factor for CKD. High rosacea severity and old age further increased CKD risk in patients with rosacea. Careful monitoring for CKD development should be included as part of integrated care for patients with rosacea.

## Introduction

Rosacea is a chronic inflammatory cutaneous disorder characterized by centrofacial erythema, telangiectasias, papules, and pustules. Aberrations in immune response and dysregulation of the neurovascular system are presumed to be key pathophysiologic components of the disease.[1, 2] Recent studies suggest that rosacea is a systemic disorder and not merely a skin condition. Prior studies reported that it is associated with dyslipidemia, hypertension, metabolic diseases, alcohol consumption, tobacco smoking, cardiovascular diseases, and gastroesophageal reflux disease,[3–5] all of which are also prevalent in patients with chronic kidney disease (CKD).[6–8]

Accumulating evidence suggests that rosacea pathogenesis is linked to overexpression of pro-inflammatory cytokines and higher reactive oxygen species production.[9–11] Similarly, previous studies reported that chronic low-grade inflammation and oxidative stress are important in CKD development.[12, 13] Because rosacea and CKD share some pathogenic mechanisms and associated conditions, it is tempting to posit an association between these diseases. Patients with inflammatory conditions such as psoriasis and rheumatoid arthritis have a high risk of CKD.[14–16] Like that of psoriasis, the underlying mechanism of rosacea is thought to be associated with inflammatory cascades.[17, 18] However, the relationship between rosacea and CKD has not been previously investigated. We therefore assessed the risk of CKD in a large, nationally representative, population-based cohort of Chinese patients with rosacea in Taiwan.

## Materials and methods

### Study design and data source

The data used in this cohort study were obtained from the Longitudinal National Health Insurance Research Database (LHID) 2000, which is a subset of the National Health Insurance Research Database (NHIRD). The NHIRD is derived from the Taiwanese National Health Insurance (NHI) program, which was launched in 1995 to finance health care for all citizens. For the LHID2000, about 1,000,000 representative individuals were randomly sampled from the NHI Registry of Beneficiaries in 2000. The database includes information on inpatient care, outpatient care, ambulatory care, and prescription drugs for the period from January 1, 1996 through December 31, 2013. And patient diagnoses were coded using the International Classification of Diseases, Ninth Revision, Clinical Modification (ICD-9-CM). The Taiwanese NHI program provides care for approximately 99% of the Taiwanese population of more than 23 million people and offers unique possibilities for research. To ensure the accuracy and reliability of coding, the Bureau of the NHI of Taiwan performs random cross-checking, requests justifications by invited physicians, imposes heavy fines for false claims and overcharging, and initiates malpractice proceedings for fraudulent claims. Thus, the NHIRD is generally regarded as accurate and reliable. Confidentiality assurances were addressed by abiding by the data regulations of the NHI Bureau, and a formal written waiver for ethical approval was obtained from the local investigational research bureau of the National Taiwan University Hospital Hsin-Chu Branch, Hsin-Chu, Taiwan (103-024-E). All patient records and information were anonymized and de-identified before the analysis.

### Study population

This retrospective cohort study analyzed data from individuals who received a new diagnosis of rosacea (ICD-9-CM code 695.3) during ambulatory visits or inpatient care episodes between January 1, 2001 and December 31, 2005. To ensure diagnostic validity, we required that patients have at least 2 dermatologist diagnoses. Because acne (ICD-9-CM code 706.1), seborrheic dermatitis (ICD-9-CM code 690.1), and cutaneous lupus erythematosus (ICD-9-CM code 695.4) are frequently confused with rosacea, patients with 2 diagnoses of any of these diseases were excluded from the study group. The initial diagnosis date was defined as the index date of entry into the rosacea cohort.

**Fig 1:**
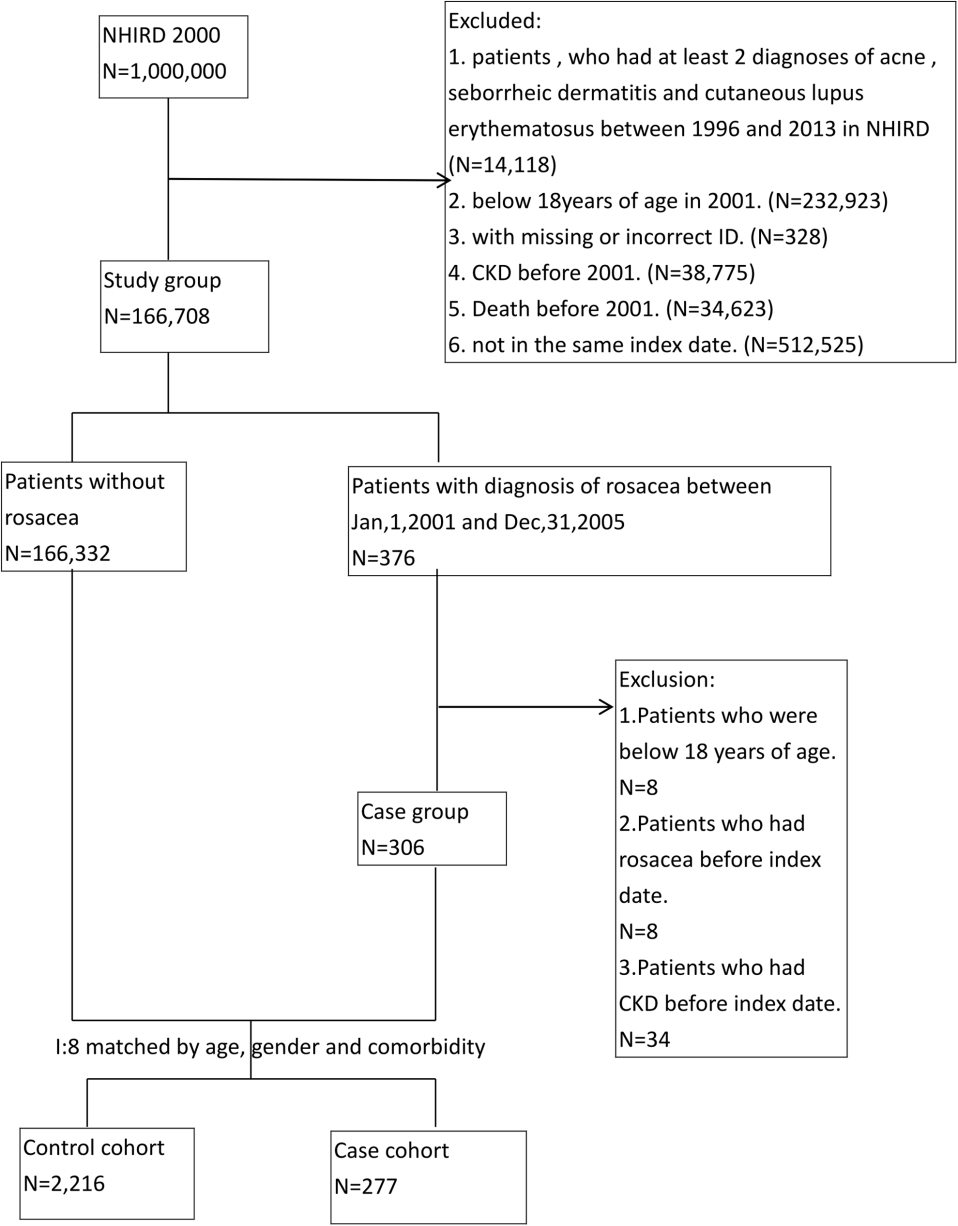
Selection of study population. CKD, chronic kidney disease; LHID, Longitudinal National Health Insurance Research Database; NHIRD, National Health Insurance Research Database.

Propensity score matching adjusted for sex, age, and comorbidities was used to assemble a comparison group among subjects without rosacea and CKD in the LHID2000. Each individual with rosacea was paired with 8 individuals without rosacea on the index enrollment date. The matched comorbidities included hypertension (ICD-9-CM codes 401–402), diabetes mellitus (ICD-9-CM 250.xx), dyslipidemia (ICD 9-CM code 272.x), and cardiovascular disease (ICD-9-CM 410–429). Patients in the study cohort and control cohort were excluded if they were younger than 18 years or had CKD or rosacea before the index date (**Fig 1**). Patients with rosacea were stratified by disease severity as having moderate-to-severe or mild rosacea. Patients who received oral drugs (including doxycycline, minocycline, tetracycline, metronidazole, and isotretinoin) for rosacea at least 3 times during the first year of follow-up were considered to have moderate-to-severe rosacea. The remaining patients were considered to have mild rosacea.

### Outcome

The primary outcome was defined as the first ambulatory visit, hospitalization, or surgical procedure for CKD (ICD-9-CM codes: 580–589, 753, 403, 404, 250.4, 274.1, 440.1, 442.1, 447.3, 572.4, 642.1, 646.2). To investigate the risk of developing CKD during the follow-up period, all individuals in the study and comparison cohorts were tracked for 8–12 years from their index enrollment date until death or the end of the study period.

### Statistical analysis

Frequencies and percentages are used to present descriptive statistics of the study sample. The chi-square test was used to compare distributions of baseline demographic characteristics and selected comorbidities between patients with and without rosacea. Cox proportional hazards regression was used to estimate the effect of rosacea on CKD. Covariates adjusted in multivariable Cox regression analysis included gender, age, diabetes, hypertension, hyperlipidemia, cardiovascular disease, systemic lupus erythematosus, rheumatoid arthritis, polycystic kidney disease, medications putatively associated with CKD risk (non-steroidal anti-inflammatory drugs, angiotensin-converting-enzyme inhibitors/angiotensin II receptor antagonists, loop diuretics, and statin), Charlson comorbidity index score, and annual ambulatory care visits, as detailed in previous studies.[19] The Kaplan–Meier method and log-rank test were used to compare cumulative incidences of CKD between patients with and without rosacea. CKD incidences in the rosacea and control cohorts were calculated by dividing the number of patients with CKD by the total number of person-years. Death before CKD incidence was considered as a competing risk event. In addition, Cox proportional hazard models with competing risk analysis were used to estimate CKD risk among rosacea patients, as described previously.[20] Analyses were performed with SAS v9.3 (SAS Institute, Cary, NC). A *P* value of <0.05 was considered to indicate statistical significance.

## Results

The study comprised 277 patients with rosacea and 2216 age-, sex-, and comorbidity-matched reference subjects. **Table 1** shows the sociodemographic characteristics of the patients. After propensity score matching, no significant differences in sex, age, or comorbidities were noted between patients with and without rosacea. Approximately 71% of patients were women and 29% were men in both the study and control groups. Most study participants were aged 30–49 years.

**Table 1:** Background characteristics and comorbidities of patients with and without rosacea.

Characteristic	No.(%) of individuals		p-value
	Patients with rosacea N = 277	Patients without rosacea N = 2216	
**Sex**			
Female	196(70.76)	1570(70.85)	0.9751
Male	81(29.24)	646(29.15)	
**Age Group**			
18–29	46(16.61)	370(16.70)	1.0000
30–39	51(18.41)	410(18.50)	
40–49	107(38.63)	857(38.67)	
50–59	43(15.52)	342(15.43)	
60+	30(10.83)	237(10.69)	
**Comorbidity**			
Diabetes	1(0.6)	8(0.36)	1.0000
Hypertension	13(4.69)	105(4.74)	0.9734
Hyperlipidemia	7(2.53)	56(2.53)	1.0000
Cardiovascular diseases	8(2.89)	52(2.35)	0.5793
Systemic lupus erythematosus	2(0.72)	2(0.09)	0.0634
Rheumatoid arthritis	2(0.72)	7(0.32)	0.2204
Polycystic kidney disease	0(0.0)	0(0.0)	_
**Charlson comorbidity index**			
0	243(87.73)	2018(91.06)	0.0512
1	28(10.11)	139(6.27)	
≧2	6(2.17)	59(2.66)	
**Drug use**	107(38.63)	906(40.88)	0.4710
Loop diuretics	1(0.36)	26(1.17)	0.3537
Statin	2(0.72)	23(1.04)	1.0000
NSAID	103(37.18)	877(39.58)	0.4423
ACEI	0(0.00)	10(0.45)	0.6144
ARB	6(2.17)	28(1.26)	0.2630
Annual ambulatory care visits^†^	19(10–29)	12(6–21)	<0.0001
<15	117(42.24)	1366(61.64)	<0.0001*
≧15	160(57.76)	850(38.36)	

^*^ p<0.05 for comparison between patients with versus without rosacea.

^†^The overall OPD times: Median (Q1-Q3) = 15(6–23) months.

**Fig 2:**
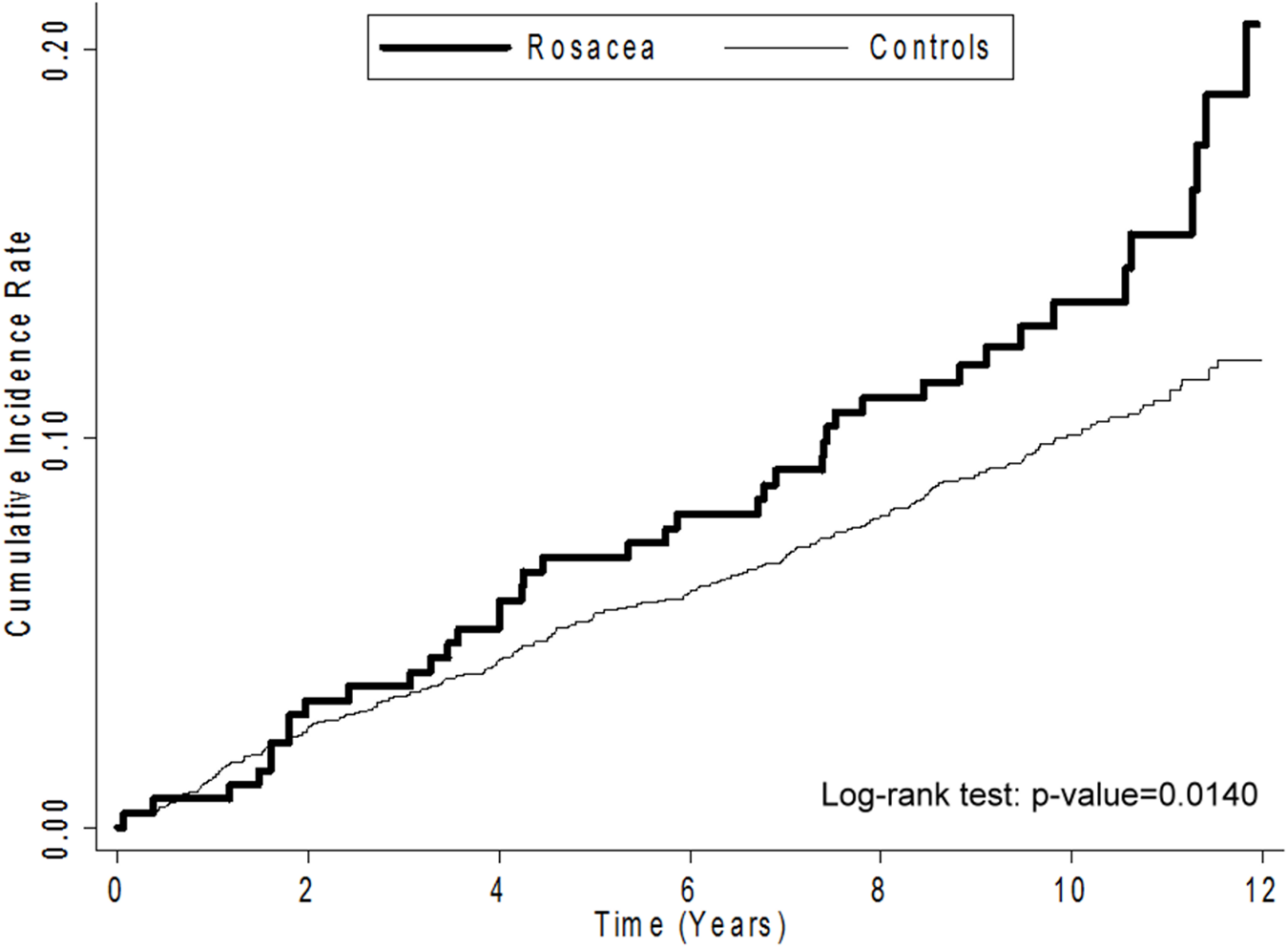
Kaplan–Meier curves. Cumulative incidence rate of CKD in patients with and without rosacea.

The median interval to CKD was 9.92 years, and there was no significant different between rosacea patients (median 9.80 years; interquartile range (IQR): 8.68–11.34) and controls (median 9.95; IQR: 8.91–11.42). The overall CKD incidence rate was higher in patients with rosacea than in the controls (16.02 vs. 10.63 per 1000 person-years, respectively). The Kaplan–Meier curves for the cumulative CKD incidence rate in patients with and without rosacea are shown in **Fig 2**. CKD development was 50% more likely in patients with rosacea (hazard ratio (HR) 1.51; 95% confidence interval (CI) 1.08–2.09) than in those without rosacea, and this association remained significant after adjusting for other covariates (adjusted HR 1.40; 95% CI 1.01–1.96). The Cox proportional hazard models with competing risk analysis yielded similar results (adjusted subdistribution (SD)-HR 2.00; 95% CI 1.05–3.82). When patients were stratified by age group, the increase in CKD risk was highest for patients aged 30–39 years. However, in the competing risk model the association between rosacea and CKD was not significant for patients aged 30–39 years. Patients older than 50 years had a significant risk of CKD (adjusted SD-HR for age 50–59 years, 3.68; 95% CI 1.00–13.56; adjusted SD-HR for 60+ years, 4.24; 95% CI 1.63–11.04) (**Table 2**).

**Table 2:** Incidences and hazard ratios for chronic kidney disease (CKD) in patients with and without rosacea.

	Patients with rosacea			Patients without rosacea			Crude HR (95%CI)	Adjusted^b^ HR (95%CI)	Crude SD-HR^c^ (95%CI)	Adjusted^b^ SD-HR^c^ (95%CI)
	CKD	PR^a^	Rate	CKD	PR^a^	Rate				
All patients	42	2622.09	16.02	229	21549.18	10.63	1.51(1.08–2.09)*	1.40(1.01–1.96)*	2.18(1.18–4.00)*	2.00(1.05–3.82)*
Gender										
Female	26	1889.54	13.76	151	15383.86	9.82	1.40(0.92–2.13)	1.41(0.93–2.15)	1.05(0.32–3.47)	1.20(0.35–4.04)
Male	16	732.55	21.84	78	6165.32	12.65	1.71(1.00–2.93)	1.41(0.81–2.47)	3.23(1.56–6.68)*	2.83(1.33–6.06)*
Age										
18–29	4	470.35	8.50	20	3734.02	5.36	1.63(0.56–4.77)	1.42(0.46–4.37)	—	—
30–39	5	482.11	10.37	12	4196.11	2.86	3.63(1.28–10.31)*	3.18(1.08–9.39)*	1.62(0.19–13.81)	1.22(0.21–7.18)
40–49	13	1081.54	12.02	70	8513.67	8.22	1.47(0.82–2.66)	1.47(0.80–2.67)	0.46(0.06–3.49)	0.72(0.08-.6.33)
50–59	10	360.72	27.72	59	3154.54	18.70	1.49(0.76–2.91)	1.41(0.71–2.81)	5.34(1.79–15.92)*	3.68(1.00–13.56)*
60+	10	227.38	43.98	68	1950.84	34.86	1.27(0.65–2.47)	1.18(0.60–2.32)	3.31(1.32–8.32)*	4.24(1.63–11.04)*

*P < 0.05.

a. per 1000 person years

b. Adjusted by the variables included in Table 1.

c. SD-HR was estimated from the competing risk model, and there is no subject death in aged at 18~29.

HR, hazard ratio; CI, confidence interval; CKD, chronic kidney disease; PY, person-years; SD-HR, sub-distribution hazard ratio.

When patients with rosacea were further categorized as those with moderate-to-severe rosacea (n = 203 (73.3%)) and those with mild rosacea (n = 74 (26.7%)). Patients with moderate-to-severe rosacea had a higher HR for CKD than those with mild rosacea. After adjustment for all other covariates and considering the competing risk of mortality, only those with moderate-to-severe rosacea had a significantly higher HR for CKD as compared with the non-rosacea controls. (SD-HR 2.53; 95% CI 1.11–5.75; *P* = 0.026) (Table 3).

**Table 3:** Hazard ratios for chronic kidney disease (CKD) by rosacea severity.

	N	CKD(%)	Crude HR (95%CI)	Adjusted^a^ HR (95%CI)	P-value	Crude SD-HR^b^ (95%CI)	Adjusted^a^ SD-HR^b^ (95%CI)	P-value
Patients without rosacea	2216	229(10.33)	Ref.	Ref.		Ref.	Ref.	
Patients with mild severity of rosacea^¶^	203	29(14.29)	1.41(0.96–2.07)	1.33(0.90–1.97)	0.1480	2.03(1.00–4.11)*	1.82(0.83–4.00)	0.1350
Patients with moderate-to-severe severity of rosacea^†^	74	13(17.57)	1.79(1.02–3.13)*	1.59(0.91–2.81)	0.1066	2.61(0.94–7.21)	2.53(1.11–5.75)	0.0266*

HR, hazard ratio; CI, confidence interval; CKD, chronic kidney disease; Ref., reference group; SD-HR, sub-distribution hazard ratio.

a. Adjusted by the variables included in Table 1.

b. SD-HR was estimated from the competing risk model.

^¶^Rosacea patients who did not receive oral medications for the disease

^†^Rosacea patients who received oral medications for the disease, including doxycycline, minocycline, tetracycline, metronidazole, and isotretinoin

*P-value<0.05.

## Discussion

Rosacea has long been considered a disease limited to the skin, but an increasing number of studies have observed an association between rosacea and extracutaneous diseases, which suggests it has far-reaching systemic effects. A recent study compared cardiovascular disease (CVD) risk factors among 60 rosacea patients and 50 age- and sex-matched controls and found that patients with rosacea were more likely than controls to have higher levels of total cholesterol (199 vs. 163 mg/dL, *P* < 0.001), low-density lipoprotein cholesterol (121 vs. 101 mg/dL, *P* = 0.002), and C-reactive protein (0.43 vs. 0.24 mg/L, *P* = 0.007) and a family history of premature CVD (*P* = 0.002).[3] Similarly, subsequent studies reported higher prevalences of insulin resistance and CVD risk factors (elevated fasting blood glucose, total cholesterol, and systolic and diastolic blood pressures, *P* < 0.05) in patients with rosacea.[4, 5, 21] Moreover, Rainer et al. suggested that rosacea is associated with increased risk of CVD,[5] and a very recent population-based study reported a significant association between rosacea and coronary artery disease.[4] These associations between rosacea and certain comorbidities do not appear to be attributable solely to shared CVD risk factors. Prior studies showed that the associations of rosacea with some comorbidities did not substantially change after adjustment for traditional CVD risk factors [4, 5, 22] and that the strength of the associations increased in relation to rosacea severity.[5, 22] These findings suggest that other factors intrinsically linked to rosacea contribute to the development of these comorbidities. Our study provided some support for the hypothesis. Most of the abovementioned CVD risk factors are shared by rosacea and CKD.[23] Nevertheless, using a propensity score matched-pair procedure and multivariable Cox proportional hazards models, we found that rosacea was significantly associated with CKD, even after adjusting for the effects of comorbidities.

Because of its increasing incidence and prevalence and progression to end-stage renal disease, CKD is a global public health burden. CKD limits longevity and increases costs to health-care systems worldwide.[24] Moreover, as compared with the general population, patients with inflammatory diseases such as rheumatoid arthritis, systemic lupus erythematosus, Sjögren syndrome, systemic scleroderma, and psoriatic disease are more likely to have CKD.[14–16, 25] Accumulating evidence indicates that chronic low-grade inflammation resulting in endothelial injury, impaired vasodilation, and glomerulosclerosis plays a major role in CKD development and that various inflammatory cytokines are related to CKD pathogenesis and progression.[12, 26–28] Rosacea is a common chronic inflammatory skin condition characterized by dysfunction in the innate and/or adaptive immune response. Pro-inflammatory cytokines such as interleukin (IL)-1 β, IL-6, IL-8, and tumor necrosis factor-α are involved in rosacea pathogenesis, and inflammasome-related genes (CASP-1 and NALP-3) are overexpressed in skin samples from rosacea patients. [9, 10, 29] These inflammatory mediators may have an important role in CKD development.[27, 30–32] Consistent with these past studies, our data indicate that patients with moderate-to-severe rosacea, who might have a greater inflammation burden, had a higher risk of developing CKD than did patients with mild rosacea. The result lends support to the hypothesis that inflammatory mediators are involved in CKD pathogenesis and progression in patients with rosacea.

Previous studies of rosacea patients reported that the activity of paraoxone-1, an antioxidant enzyme, was decreased and that oxidative stress was increased.[33] In addition, there was a significant positive correlation between the magnitude of the reduction in cutaneous antioxidant capacity and rosacea severity, indicating that oxidative stress may have a role in the pathophysiology of rosacea.[33, 34] Similarly, evidence from numerous clinical studies confirms the importance of oxidative stress in CKD, as it can trigger the inflammatory process and accelerate progression of renal injury.[13, 35] Although the evidence is not conclusive, mechanisms involving inflammatory pathways and oxidative stress appear to contribute to the association between rosacea and CKD risk.

The present competing risk model showed a significant association between rosacea and CKD for patients older than 50 years, and CKD risk was highest among adults older than 60 years. A possible explanation for these findings is that age-associated decline in kidney function and loss of renal functional reserve in elderly adults increases the effects of rosacea-induced inflammation and oxidative stress on CKD development. Nevertheless, more research is needed in order to confirm this hypothesis.

Although this study was based on a large, high-quality, nationwide, population-based database, several limitations must be considered. First, diagnosis of rosacea was based on secondary claims data, and misclassification is thus possible. To minimize this bias, the presence of disease was defined as at least 2 ICD-9-CM diagnoses made by a relevant specialist. Moreover, the reliability and validity of using claims data to identify patients with rosacea and CKD have been demonstrated in previous epidemiologic studies.[4, 14–16, 36–38] This study relied on ICD diagnoses for identification of rosacea and CKD. It is likely that some asymptomatic patients did not seek medical treatment and therefore were not captured by this study design. Some misclassification of patients with acute kidney injury or without CKD may have occurred in the rosacea and control groups. However, this misclassification would have occurred in both the rosacea and control groups, would thus be nondifferential, and would bias effect estimates toward the null. Second, the fact that hospital visits are more frequent for patients with rosacea than for the general population may result in potential surveillance bias. However, laboratory testing of serum creatinine concentration and urine analysis, which are required for CKD diagnosis, were not routinely performed during therapeutic monitoring of rosacea. Moreover, the present results remained robust after adjustment for number of doctor visits in both groups. Third, the NHIRD did not include information on rosacea subtypes, lifestyle factors, or laboratory findings. Thus, we were unable to estimate glomerular filtration rate or detect proteinuria at baseline and could not adjust for these unmeasured potential confounders. Fourth, we used treatment with systemic therapies as a surrogate marker for severe disease. Doxycycline, minocycline, and tetracycline are also used to treat acne vulgaris and some infectious diseases. However, the probability of misclassification within this specific cohort with an ICD diagnosis of rosacea is likely to be negligible. Lastly, the present Taiwanese study population mainly consisted of Taiwanese with Han ethnicity and mostly type III/IV Fitzpatrick skin types.[39] Therefore, caution is advised when attempting to extrapolate our results to patients of other ethnicities.

In conclusion, the present results indicate that patients with rosacea, particularly those with moderate-to-severe disease, have an increased risk of CKD that is not completely explained by traditional risk factors. Future research should attempt to identify the mechanisms underlying this increase in CKD risk. Patients with rosacea and their physicians should be aware of this potential link with CKD. Careful monitoring of renal function and avoidance of long-term use of nephrotoxic drugs should be considered as part of integrated care for patients with rosacea, particularly those older than 50 years.
